# Explore! Dream! Discover!

**DOI:** 10.3325/cmj.2018.59.1

**Published:** 2018-02

**Authors:** Ana Marušić, Matko Marušić

**Affiliations:** University of Split School of Medicine, Split, Croatia *ana.marusic@mefst.hr*

The last time we wrote for the *Croatian Medical Journal* (CMJ) was in 2009 (1) and 2011 (2), when we celebrated the CMJ’s 20th anniversary. After creating and editing CMJ for twenty years, it was time to let “our child” take its own way into the world. Our advice for this challenging but thrilling voyage is best expressed by Mark Twain’s quote (2): “Twenty years from now you will be more disappointed by the things that you didn’t do than by the ones you did do. So throw off the bowlines. Sail away from the safe harbor. Catch the trade winds in your sails. Explore. Dream. Discover.”

In December 2017, we were invited to celebrate the CMJ’s 25th anniversary and to see how it sails on. What impressed us most was that the journal has the best people for the job: the editorial team has some old members, who have been with the journal almost from its beginning and who can provide continuity and editorial wisdom, and new young editors, who can take on the challenge of the rapidly developing world of scientific communication and editing.

The silver anniversary was also a time to remind us all why we had started the journal (3) – to have the Croatian name in a newly independent Croatia, to open the doors for the Croatian medical research to join the global scientific community and the window to the global community to see Croatian research, and to foster research excellence and research integrity in the Croatian academic community. Our strategic approach to these goals was openness and transparency, international visibility, originality of research published, and volunteer work of the editors to ensure independence and integrity. We achieved these goals through a clear definition of work in the journal, educational role of the editors, author-helpful policy, active soliciting of articles, taking journal as a research model, and involvement of young academics in journal work and research (3,4). Through our mentoring work, we not only built CMJ into an internationally successful, respected, and influential journal, but we also helped to publish many research articles outside CMJ, introduced mandatory courses on research methodology at all medical schools in Croatia, introduced international publishing standards and innovations into the journal, and helped other Croatian journals to reach international visibility and excellence (3-6).

During the five years after we had left, CMJ continued to publish good science, kept its bibliometric indices at the same high levels, and trained new editors. Is this enough? We would like to repeat our challenge to the CMJ’s new editor-in-chief and his team (2), only this time using exclamation marks: Explore! Dream! Discover! For a journal to stay at the forefront of science, both globally and locally, it is important to innovate, to not only face but create challenges in order to grow and mature, and to lead rather than follow (7). For a journal, the greatness is preserved by constant innovation and change.

CMJ has a great potential for such change. Even during our editorship, the journal moved from topics related to social aspects of medicine, including the consequences of natural and man-made disasters such as war (3), toward forensic DNA science and personalized medicine (8). CMJ continues to be bibliographically related to forensic science journals in terms of citation relatedness statistics and to serve the expanding and productive community of translational medicine. Predictive, preventive, personalized, and participatory (P4) medicine could be the new challenge for CMJ, combining it with its important national commitment.

Regardless of its future, the focus of the journal should always include its responsibility to the society, just as social responsibility is at the core of the Europe’s Horizon 2020 program (9). We should not forget how we responded to social challenges during the Homeland War and how CMJ helped to document the extraordinary work of health professionals to alleviate the war traumas (10) (Figure 1). CMJ should continue to actively participate in societal challenges and allow the dialogue about the problems that need change to improve the health and well-being of the society. As Hugh Clegg, the former editor of the BMJ, said about the role of medical journals: “A subject that needs reform should be kept before the public until it demands reform.”

**Figure 1:**
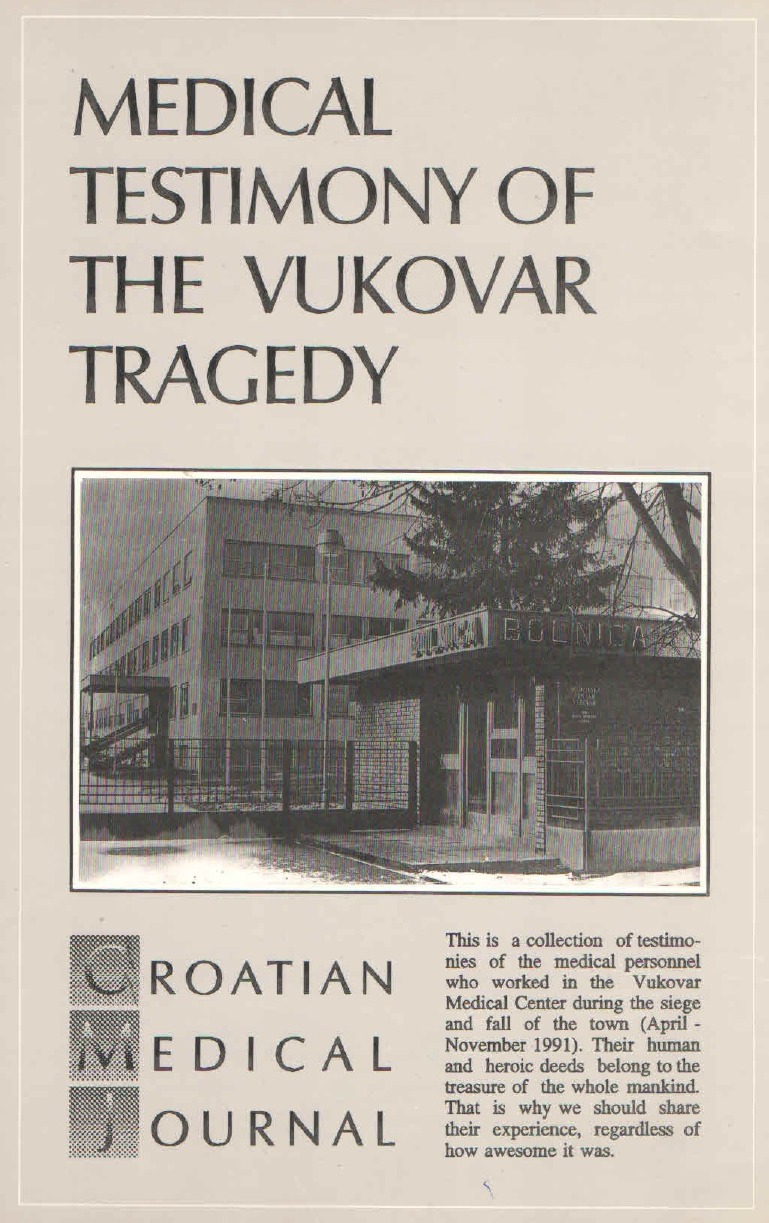
Medical Testimony of the Vukovar Tragedy is a collection of testimonies from the staff of the Vukovar Hospital, which the editors of *Croatian Medical Journal* and colleague physicians collected within a month after the fall of the city of Vukovar in November 1991. The book was published on the Human Rights Day, the 10th December 1991.

## References

[1] Marušić M (2009). Conflict of interest for editor: sweet and sad choices.. Croat Med J.

[2] Marušić A, Damjanov I (2011). CMJ’s twentieth anniversary: excellence for the future.. Croat Med J.

[3] Marušić A, Marušić M (2012). Can small journals provide leadership?. Lancet.

[4] Marušić M, Bošnjak D, Rulic-Hren S, Marušić A (2003). Legal regulation of the *Croatian Medical Journal*: model for small academic journals.. Croat Med J.

[5] Marušić M, Markulin H, Lukić IK, Marušić A (2006). Academic advancement of authors receiving tutoring from a medical journal.. Teach Learn Med.

[6] Marušić M (2012). May the wind be at your back.. Trans Marit Sci..

[7] Smith R (2005). Can medical journals lead or must they follow?. Med J Aust.

[8] Marušić A (2009). Morphology: bodies, genes, journals.. Croat Med J.

[9] European Commission. Science with and for society. Available: https://ec.europa.eu/programmes/horizon2020/en/h2020-section/science-and-society. Accessed: February 1, 2018.

[10] Marušić M, Petrovečki M, editors. Medical testimony of the Vukovar tragedy. Zagreb: Crotian Medical Journal and Medicinska naklada; 1991.

